# Plasma progastrin‐releasing peptide level shows different predictive profiles for treatment response by androgen receptor axis‐targeted agents in patients with metastatic castration‐resistant prostate cancer

**DOI:** 10.1002/cnr2.1762

**Published:** 2022-12-05

**Authors:** Masahiro Yashi, Daisaku Nishihara, Megumi Yokoyama, Hirotaka Fuchizawa, Akihito Okazaki, Kohei Takei, Issei Suzuki, Kazumasa Sakamoto, Toshiki Kijima, Minoru Kobayashi, Takao Kamai

**Affiliations:** ^1^ Department of Urology Dokkyo Medical University Tochigi Japan; ^2^ Department of Urology Utsunomiya Memorial Hospital Tochigi Japan

**Keywords:** androgen receptor axis‐targeted agents, castration‐resistant prostate cancer, gastrin‐releasing peptide, neuroendocrine pathway, progastrin‐releasing peptide

## Abstract

**Background:**

The neuroendocrine (NE) pathway cannot be ignored as a mechanism for castration‐resistant prostate cancer (CRPC) progression. The neuromediator, gastrin‐releasing peptide (GRP) may be involved in the aberrant activation of the normal androgen receptor (AR) and increased AR variants. This study focused on plasma levels of progastrin‐releasing peptide (ProGRP) and examined the treatment outcomes with androgen receptor axis‐targeted (ARAT) agents.

**Methods:**

One hundred patients with metastatic CRPC were enrolled. Enzalutamide (ENZ) or abiraterone acetate/prednisone (AA/P) were administered to 50 patients each in a nonrandomized manner as a first‐line or later choice. Plasma ProGRP levels were determined using a chemiluminescent enzyme immunoassay, and data were collected prospectively. The study endpoints were prostate‐specific antigen (PSA) response and survival estimates.

**Results:**

In the ENZ series, ProGRP levels correlated with the maximum PSA change from baseline (high ProGRP: −34.5% vs. low ProGRP: −85.7% *p* = .033). PSA progression‐free survival (PFS), radiographic/symptomatic (r/s) PFS, and overall survival (OS) in patients with high ProGRP were significantly worse than those in patients with low ProGRP (median PSA‐PFS: 3.3 vs. 10.0 months, *p =* .001, r/s PFS: 5.0 vs. 15.0 months, *p* < 0.001, and OS 17.5 vs. 49.0 months, *p* < .001, respectively). In addition, ProGRP showed an independent predictive value for all survival estimates in multivariate analyses. In the AA/P series, ProGRP levels did not correlate with the PSA change or predict PSA‐PFS and r/s PFS, but they maintained a significant difference in OS (19.0 vs. 48.0 months, *p* = .003).

**Conclusions:**

Plasma ProGRP provides a consistent predictive value for OS in metastatic CRPC patients who underwent therapy with ARAT agents. Meanwhile, ProGRP showed different predictive profiles for PSA‐ and r/s PFS between ENZ and AA/P. These findings clinically suggest a mechanism for CRPC progression involving the NE pathway via the GRP. The underlying mechanism of different predictive profiles by the ARAT agent should be explored in future research.

## 1 BACKGROUND

Prostate cancer (PCa) has become the most prevalent malignant disease and the sixth leading cause of death in Japanese men.^1^ Androgen deprivation therapy (ADT), represented by surgical or medical castration, has been the mainstay treatment for metastatic PCa for more than 70 years. However, PCa, an extremely heterogeneous disease from a phenotypic and genetic perspective, eventually develops resistance to ADT. The upfront use of docetaxel chemotherapy or treatments with androgen receptor axis‐targeted (ARAT) agents in addition to ADT is a recent trend resulting in prolonged survival in metastatic PCa patients characterized as having castration‐sensitive and high‐risk cancer.^2^ Furthermore, poly (ADP‐ribose) polymerase (PARP) inhibitors have been shown to prolong the survival of metastatic castration‐resistant PCa (CRPC) patients with germline/somatic BRCA gene mutations or other genomic alterations in DNA damage repair and are attracting attention as a precursor to personalized treatment using genomic analyses.^3^, ^4^

Most mechanisms for CRPC progression involve androgen receptor (AR) signaling.^5^ Although there is still room for improvement in treatment, the recent development of ARAT agents, their combined use, and a sequence that maximizes the therapeutic effect have offered clues to an optimal solution. However, there remains an unmet need to develop treatments targeting mechanisms other than AR signaling.^6^ Furthermore, the mechanisms for CRPC progression are probably not only independent of each other but also interact with AR signaling.^7^

Despite the long‐standing debate about the pros and cons, the neuroendocrine (NE) pathway has been recognized as one of CRPC progression mechanisms that cannot be ignored.^8^, ^9^ Gastrin‐releasing peptide (GRP) is a neuropeptide consisting of 27 amino acids produced by NE tissue.^10^ The majority of human prostate cancers express bombesin/GRP receptors, implying that GRP‐mediated regulation is involved in PCa progression.^11^ Moreover, basic research using a conventional PCa cell line, such as lymph node carcinoma of the prostate (LNCaP), suggests that GRP aberrantly activates normal AR in the absence of androgen and is involved in increasing AR‐variants by activating nuclear transcription signaling.^12^, ^13^ Hence, the mechanisms of disease progression involving the NE pathway likely extend to nonsubtype adenocarcinoma, as well as to small‐cell NE carcinoma.

This study focused on the correlation of the plasma level of progastrin‐releasing peptide (ProGRP), a bioactive precursor of GRP, with the treatment response and survival rate in patients with metastatic CRPC after administration of ARAT agents. Furthermore, we discuss the involvement of the NE pathway as a possible mechanism of CRPC progression.

## 2 PATIENTS AND METHODS

### 2.1 Patient enrollment

One hundred consecutive patients with metastatic CRPC who received oral ARAT agents at our prostate center were enrolled in this study. CRPC status was confirmed by two or more consecutive elevations of PSA levels 4 weeks apart and serum testosterone less than 50 ng/dl. All patients continued on luteinizing hormone‐releasing hormone agonists or antagonists during the study. The criteria for enrollment indicated that the patient's condition had not deteriorated seriously. An Eastern Cooperative Oncology Group performance status (ECOG‐PS) of two or less with a life expectancy of more than 6 months and adequate functions of vital organs was used to evaluate the patient's condition for inclusion.

Forty‐seven patients had bone‐only metastases, and 53 had bone plus lymph nodes or lung/liver metastases (Table 1). Patients diagnosed with NE cancer and those with only lymph node metastases (M1a) were excluded from this study.

**TABLE 1:** Patient characteristics before treatment with ARAT agents

	Median (IQR) or number		*p* value
Factor	ENZ (*n* = 50)	AA/P (*n* = 50)	
Age (years)	77 (71–82)	74 (70–81)	.186
ECOG PS 0/≥1	36/14	36/14	1.000
Hemoglobin (g/dl)	12.1(11.0–12.9)	12.3 (10.8–12.9)	.743
NLR ratio	2.7 (2.1–4.2)	2.5 (1.6–4.1)	.505
Testosterone (ng/dl)	3.0 (3.0–8.3)	3.0 (3.0–9.0)	.860
PSA (ng/ml)	14.6 (4.5–89.2)	6.6 (2.5–39.5)	.107
ALP (IU/l)	283.5 (196.8–487.0)	307.5 (204.3–382.8)	.901
NSE (ng/ml)	12.4 (9.5–15.4)	11.3 (9.9–14.9)	.858
ProGRP (pg/ml)	59.7 (48.5–80.9)	53.0 (41.0–71.7)	.208
Duration 1st ADT (month)	15.5 (9.3–24.0)	16.5 (9.0–26.5)	.720
Dx to ARAT agent (month)	34.0 (21.0–78.0)	30.0 (16.3–63.3)	.255
Prior docetaxel (*n*)	13	12	1.000
Prior ARAT agent (*n*)	12	17	.378
Bone plus/Bone only (*n*)	25/25	28/22	.689
Follow‐up period (month)	26.5 (12.3–48.0)	24.0 (14.3–45.3)	.704

Abbreviations: ARAT, androgen receptor‐axis‐targeted; ENZ, enzalutamide; AA/P, abiraterone acetate/prednisone; IQR, interquartile range; ECOG PS, Eastern Cooperative Oncology Group performance status; NLR, neutrophil to lymphocyte ratio; PSA, prostate‐specific antigen; ALP, alkaline phosphatase; NSE, neuron‐specific enolase; ProGRP, progastrin‐releasing peptide; Dx, diagnosis; ADT, androgen‐deprivation therapy.

### 2.2 ARAT agents and treatment history

A standard dose of enzalutamide (ENZ) or abiraterone acetate/prednisone (AA/P) was orally administered to 50 patients each in a nonrandomized manner for CRPC treatment. The treatment was continued until radiographic or symptomatic progression was confirmed or unacceptable toxicity occurred. The median period of the primary ADT was 16.0 (interquartile range 9 to 25) months, and the median interval of the initial PCa diagnosis to study entry was 33.0 months. ARAT agents were administered as first‐line treatment in 28 and 26 patients in the ENZ and AA/P series, respectively. In the ENZ series, 13 patients were exposed and resistant to docetaxel‐based chemotherapy (DOC), 12 to AA/P, and 3 to both treatments. In the AA/P series, 12 patients were exposed and resistant to DOC, 17 to ENZ, and 5 to both treatments (Table 1). These patients did not overlap between the ENZ and AA/P series.

### 2.3 ProGRP and other laboratory tests

Laboratory data were prospectively recorded in a computer database in a stand‐alone environment. Plasma ProGRP (Lumipulse® G ProGRP, Fujirebio, Tokyo, Japan) and serum neuron‐specific enolase (NSE) (ECLusys® reagent NSE, Roche Diagnostics, Tokyo, Japan) levels were measured every 6 months or when a unique event occurred. The other laboratory tests were conducted monthly, including prostate‐specific antigen (PSA) (Atellica® IM cPSA, Siemens Healthineers, Tokyo, Japan). No differences in patient characteristics and laboratory data were determined between the ENZ and AA/P series prior to ARAT agent administration (Table 1).

### 2.4 Interpretation of plasma ProGRP values

The biomarker, ProGRP, was developed to find small‐cell lung cancer and has become widely used as a marker for diagnosis and clinical follow‐up of cancers with NE characteristics.^14^, ^15^ Since October 2011, ProGRP, which was measured in serum by enzyme‐linked immunosorbent assay, has been changed to plasma measurement using a chemiluminescent enzyme immunoassay. Plasma ProGRP has higher storage stability than serum. However, prompt measurement is recommended as the value decreases by around 10% in frozen specimens over several days. The cut‐off values of 46 pg/mL in serum and 81 pg/mL in plasma were determined to distinguish small‐cell lung cancer from other histological types and benign diseases. Therefore, when interpreting ProGRP levels in PCa patients, it is necessary to understand that they are scaled down in proportion to NE tissue components compared to a pure histologic type of small‐cell carcinomas, such as lung or prostate.

### 2.5 PSA response and survival analyses

The primary endpoint was PSA response to treatment, including the percentage of change in PSA from baseline to 12 weeks and the maximum change in PSA after treatment, as recommended in the Prostate Cancer Clinical Trials Working Group 2.^16^ The secondary endpoint was survival estimates, including PSA progression‐free survival (PFS), radiographic and/or symptomatic (r/s) PFS, and overall survival (OS). PSA‐PFS was defined as the time from enrollment in the study until 25% or greater and 2 ng/ml increases in PSA from the nadir level. r/s PFS was defined as a worsening of disease‐related symptoms and/or radiographic changes. Radiographic changes included a size increase of an existing lesion or the appearance of new metastatic lesions. OS was defined as the time from enrollment in the study until death from any cause.

### 2.6 Statistical analyses

The cut‐off levels for each factor were determined as the median values of patients in the ENZ series or the appropriate grouping. To minimize the loss of statistical information due to dichotomies for continuous variables, time‐dependent receiver operating characteristic curves were not used to determine the cut‐off values. Quantitative and qualitative data were compared using the Mann–Whitney U‐test and Fisher's exact test, respectively. Survival was estimated using a Kaplan–Meier analysis, and differences were compared with the log‐rank test. A Cox regression analysis was used to estimate the hazard ratio (HR) with a 95% confidence interval (CI).

The requirement for factors to be included in the multivariate analyses was that they were significant, or showed a significant trend, in the univariate analysis and be predicted factors in previous studies. In addition, the number of events was considered and the analysis was performed using an appropriate model.

All statistical analyses were performed using EZR, a graphical user interface for R (2020 The R Foundation for Statistical Computing, version 4.0.3). All statistical tests were two‐sided, with a *p*‐value of less than 0.05 considered to be statistically significant.

### 2.7 Ethics and patient consent

This study was conducted in accordance with the Helsinki Declaration and was approved by our institutional ethical review boards (approval number #28010, including the responsibility to report the results of this study). In addition, each patient signed a consent form regarding the storage of their information for the purpose of research and acknowledged that the results of this study would not affect their subsequent clinical course.

## 3 RESULTS

### 3.1 PSA response and survival after enzalutamide

**FIGURE 1:**
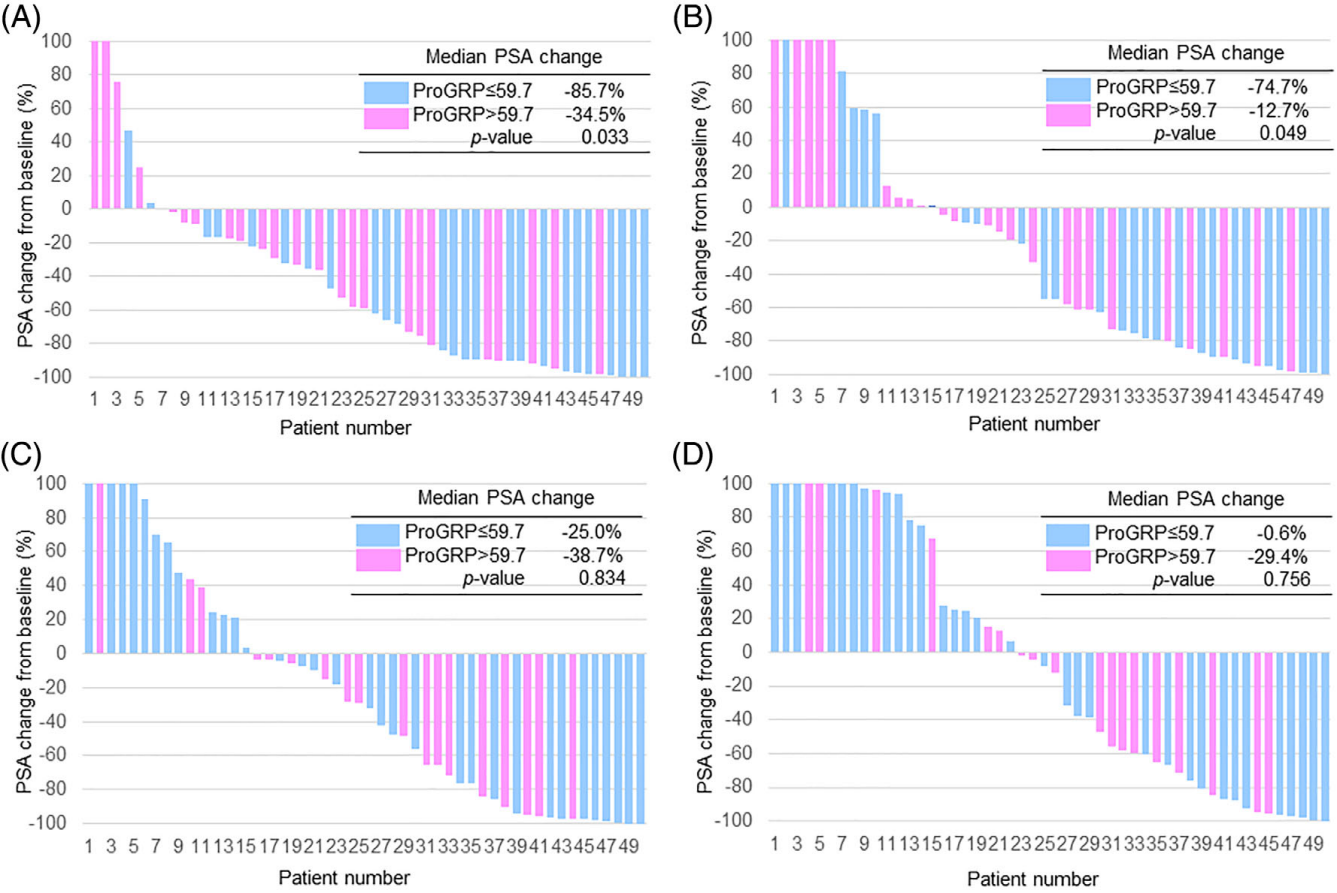
Waterfall plot showing PSA changes at maximum (A) and 12 weeks (B) from baseline after treatment with enzalutamide, and those at maximum (C) and 12 weeks (D) from baseline after treatment with abiraterone acetate/prednisone.

In the ENZ series, PSA responses were compared between two groups divided by the median plasma ProGRP value of 59.7 pg/ml. The plasma ProGRP levels correlated with the maximum PSA change from baseline (high ProGRP: −34.5% vs. low ProGRP: −85.7%, *p* = .033), and PSA change at 12 weeks after treatment (−12.7% vs. −74.7%, *p* = .049) (Figure 1A,B).

**FIGURE 2:**
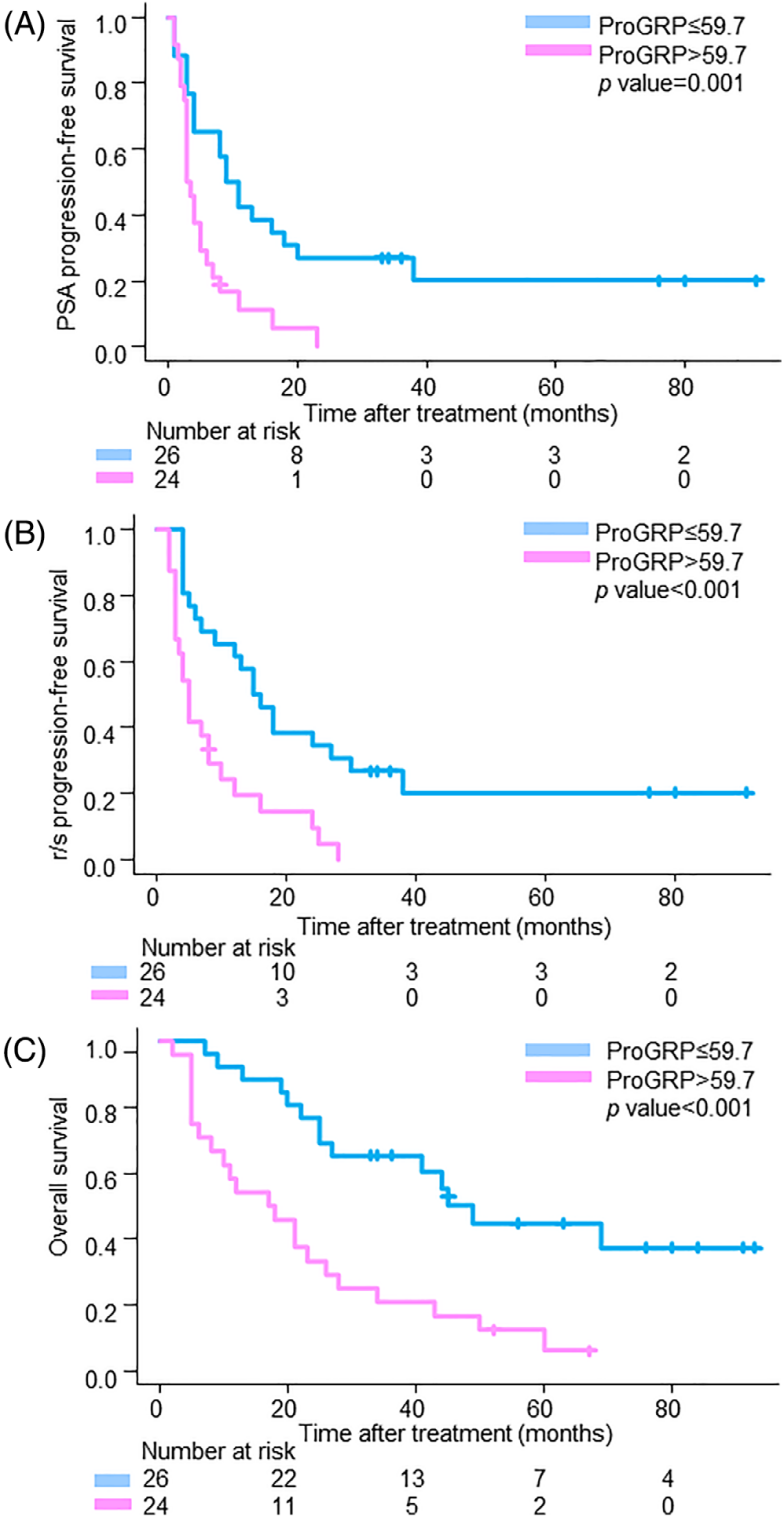
Kaplan–Meier curves of survival outcomes for metastatic CRPC patients who underwent treatment with enzalutamide (A: PSA‐PFS, B: r/s PFS, C: OS).

After a median follow‐up of 26.5 months, 43 (86.0%) patients were interpreted as having PSA and r/s progression, and 36 (72.0%) patients had died. PSA‐PFS, r/s PFS, and OS in patients with high ProGRP were significantly worse than those in patients with low ProGRP (median PSA‐PFS: 3.3 months vs. 10.0 months, *p =* .001, r/s PFS: 5.0 months vs. 15.0 months, *p* < .001, and OS: 17.5 months vs. 49.0 months, *p* < .001, respectively) (Figure 2A–C).

In the Cox regression analyses for PSA‐PFS, the univariate analysis showed that ProGRP, duration of primary ADT, and prior use of AA/P were statistically significant factors, and multivariate analyses showed that ProGRP and the prior use of AA/P sustained their independent predictive value (ProGRP: HR 2.54, 95% CI 1.31–4.92, *p* = .006) (Table 2). In analyses for r/s PFS, the univariate analysis showed that ProGRP, duration of primary ADT, and prior use of AA/P were statistically significant factors, and multivariate analyses indicated their independent predictive value and that of alkaline phosphatase (ALP) (ProGRP: HR 3.04, 95% CI 1.58–5.83, *p* < .001) (Table 3). In the OS analyses, the univariate analysis showed that ProGRP, ECOG‐PS, hemoglobin, PSA, and ALP were statistically significant factors. In the multivariate analyses, ProGRP, ALP, and ECOG‐PS had independent predictive values (ProGRP: HR 2.92, 95% CI 1.30–6.53, *p* = .009) (Table 4).

**TABLE 2:** Factors associated with PSA progression‐free survival after treatment with ENZ

		Univariate			Multivariate^a^		
Factor	Category (*n*)	HR	95% CI	*p*‐value	HR	95% CI	*p*‐value
Age (years)	>77 (22) vs. ≤77 (28)	1.177	0.639–2.166	.601	—	—	—
ECOG PS 0/≥1	≥1 (14) vs. 0 (36)	1.558	0.796–3.051	.196	—	—	—
Hemoglobin (g/dl)	≤12.1 (25) vs. >12.1(25)	1.872	0.988–3.546	.054	—	—	—
NLR ratio	>2.7 (25) vs. ≤2.7 (25)	1.513	0.804–2.850	.200	—	—	—
Testosterone (ng/dl)	≤3.0 (30) vs. >3.0 (20)	1.592	0.830–3.079	.167	—	—	—
PSA (ng/ml)	>14.6 (25) vs. ≤14.6 (25)	1.287	0.706–2.346	.410	—	—	—
ALP (IU/l)	>284 (24) vs. ≤284 (26)	1.376	0.752–2.518	.301	—	—	—
NSE (ng/ml)	>12.4 (24) vs. ≤12.4 (26)	1.321	0.709–2.463	.381	—	—	—
ProGRP (pg/ml)	>59.7 (24) vs. ≤59.7 (26)	2.620	1.383–4.964	.003	2.535	1.307–4.917	.006
Duration 1st ADT (month)	≤15.5 (26) vs. >15.5 (24)	1.973	1.067–3.648	.030	1.863	0.979–3.544	.058
Dx to ENZ (month)	≤34.0 (25) vs. >34.0 (25)	1.003	0.548–1.837	.991	—	—	—
Prior docetaxel	yes (13) vs. no (37)	1.450	0.749–2.807	.270	—	—	—
Prior AA/P	yes (12) vs. no (38)	2.574	1.282–5.168	.008	3.227	1.583–6.578	.001
Metastatic burden	Bone+ (25) vs. Bone only (25)	1.282	0.699–2.352	.423	—	—	—

^a^ Analysis model consisted of appropriate factors.

Abbreviations: AA/P, abiraterone acetate/prednisone; ADT, androgen‐deprivation therapy; ALP, alkaline phosphatase; CI, confidence interval; Dx, diagnosis; ECOG PS, Eastern Cooperative Oncology Group performance status; ENZ, enzalutamide; HR, hazard ratio; NLR, neutrophil to lymphocyte ratio; NSE, neuron‐specific enolase; ProGRP, progastrin‐releasing peptide; PSA, prostate‐specific antigen; vs, versus.

**TABLE 3:** Factors associated with r/s progression‐free survival after treatment with ENZ

		Univariate			Multivariate^a^		
Factor	Category (*n*)	HR	95% CI	*p*‐value	HR	95% CI	*p*‐value
Age (years)	>77 (22) vs. ≤77 (28)	1.178	0.642–2.157	.597	—	—	—
ECOG PS 0/≥1	≥1 (14) vs. 0 (36)	1.538	0.782–3.023	.212	—	—	—
Hemoglobin (g/dl)	≤12.1 (25) vs. >12.1(25)	1.856	0.990–3.481	.054	—	—	—
NLR ratio	>2.7 (25) vs. ≤2.7 (25)	1.625	0.867–3.046	.130	—	—	—
Testosterone (ng/dl)	≤3.0 (30) vs. >3.0 (20)	1.386	0.719–2.673	.330	—	—	—
PSA (ng/ml)	>14.6 (25) vs. ≤14.6 (25)	1.474	0.807–2.692	.207	—	—	—
ALP (IU/l)	>284 (24) vs. ≤284 (26)	1.787	0.973–3.282	.062	3.749	1.719–8.174	<.001
NSE (ng/ml)	>12.4 (24) vs. ≤12.4 (26)	1.464	0.784–2.732	.231	—	—	—
ProGRP (pg/ml)	>59.7 (24) vs. ≤59.7 (26)	2.873	1.520–5.430	.001	3.038	1.583–5.834	<.001
Duration 1st ADT (month)	≤15.5 (26) vs. >15.5 (24)	1.908	1.035–3.516	.038	3.616	1.653–7.913	.001
Dx to ENZ (month)	≤34.0 (25) vs. >34.0 (25)	1.017	0.556–1.860	.956	—	—	—
Prior docetaxel	yes (13) vs. no (37)	1.358	0.705–2.618	.360	—	—	—
Prior AA/P	yes (12) vs. no (38)	2.614	1.255–5.443	.010	3.932	1.823–8.484	<.001
Metastatic burden	Bone+ (25) vs. Bone only (25)	1.201	0.655–2.202	.554	—	—	—

^a^ Analysis model consisted of appropriate factors.

Abbreviations: AA/P, abiraterone acetate/prednisone; ADT, androgen‐deprivation therapy; ALP, alkaline phosphatase; CI, confidence interval; Dx, diagnosis; ECOG PS, Eastern Cooperative Oncology Group performance status; ENZ, enzalutamide; HR, hazard ratio; NLR, neutrophil to lymphocyte ratio; NSE, neuron‐specific enolase; ProGRP, progastrin‐releasing peptide; PSA, prostate‐specific antigen; r/s, radiographic and/or symptomatic; vs, versus.

**TABLE 4:** Factors associated with overall survival after treatment with ENZ

		Univariate			Multivariate^a^		
Factor	Category (*n*)	HR	95% CI	*p*‐value	HR	95% CI	*p*‐value
Age (years)	>77 (22) vs. ≤77 (28)	1.373	0.711–2.651	.345	—	—	—
ECOG PS 0/≥1	≥1 (14) vs. 0 (36)	3.761	1.857–7.617	<.001	3.256	1.424–7.444	.005
Hemoglobin (g/dl)	≤12.1 (25) vs. >12.1(25)	2.160	1.104–4.226	.024	—	—	—
NLR ratio	>2.7 (25) vs. ≤2.7 (25)	1.541	0.781–3.040	.212	—	—	—
Testosterone (ng/dl)	≤3.0 (30) vs. >3.0 (20)	1.150	0.567–2.332	.698	—	—	—
PSA (ng/ml)	>14.6 (25) vs. ≤14.6 (25)	3.335	1.672–6.652	<.001	1.587	0.595–4.235	.356
ALP (IU/l)	>284 (24) vs. ≤284 (26)	2.718	1.377–5.363	.004	2.616	1.086–6.302	.032
NSE (ng/ml)	>12.4 (24) vs. ≤12.4 (26)	1.792	0.897–3.582	.099	1.699	0.799–3.615	.169
ProGRP (pg/ml)	>59.7 (24) vs. ≤59.7 (26)	3.223	1.611–6.445	<.001	2.917	1.304–6.525	.009
Duration 1st ADT (month)	≤15.5 (26) vs. >15.5 (24)	1.652	0.849–3.213	.139	—	—	—
Dx to ENZ (month)	≤34.0 (25) vs. >34.0 (25)	1.308	0.679–2.521	.422	—	—	—
Prior docetaxel	yes (13) vs. no (37)	1.082	0.532–2.202	.828	—	—	—
Prior AA/P	yes (12) vs. no (38)	1.481	0.713–3.079	.292	—	—	—
Metastatic burden	Bone+ (25) vs. Bone only (25)	0.848	0.440–1.635	.623	—	—	—

^a^ Analysis model consisted of appropriate factors.

Abbreviations: AA/P, abiraterone acetate/prednisone; ADT, androgen‐deprivation therapy; ALP, alkaline phosphatase; CI, confidence interval; Dx, diagnosis; ECOG PS, Eastern Cooperative Oncology Group performance status; ENZ, enzalutamide; HR, hazard ratio; NLR, neutrophil to lymphocyte ratio; NSE, neuron‐specific enolase; ProGRP, progastrin‐releasing peptide; PSA, prostate‐specific antigen; r/s, radiographic and/or symptomatic; vs, versus.

### 3.2 PSA response and survival after abiraterone acetate/prednisone

In the AA/P series, plasma ProGRP levels did not correlate with PSA change after treatment (PSA change at the maximum and at 12 weeks were high ProGRP: −38.7% vs. low ProGRP: −25.0%, *p* = .834, and high ProGRP: −29.4% vs. low ProGRP: −0.6%, *p* = .756, respectively) (Figure 1C,D).

**FIGURE 3:**
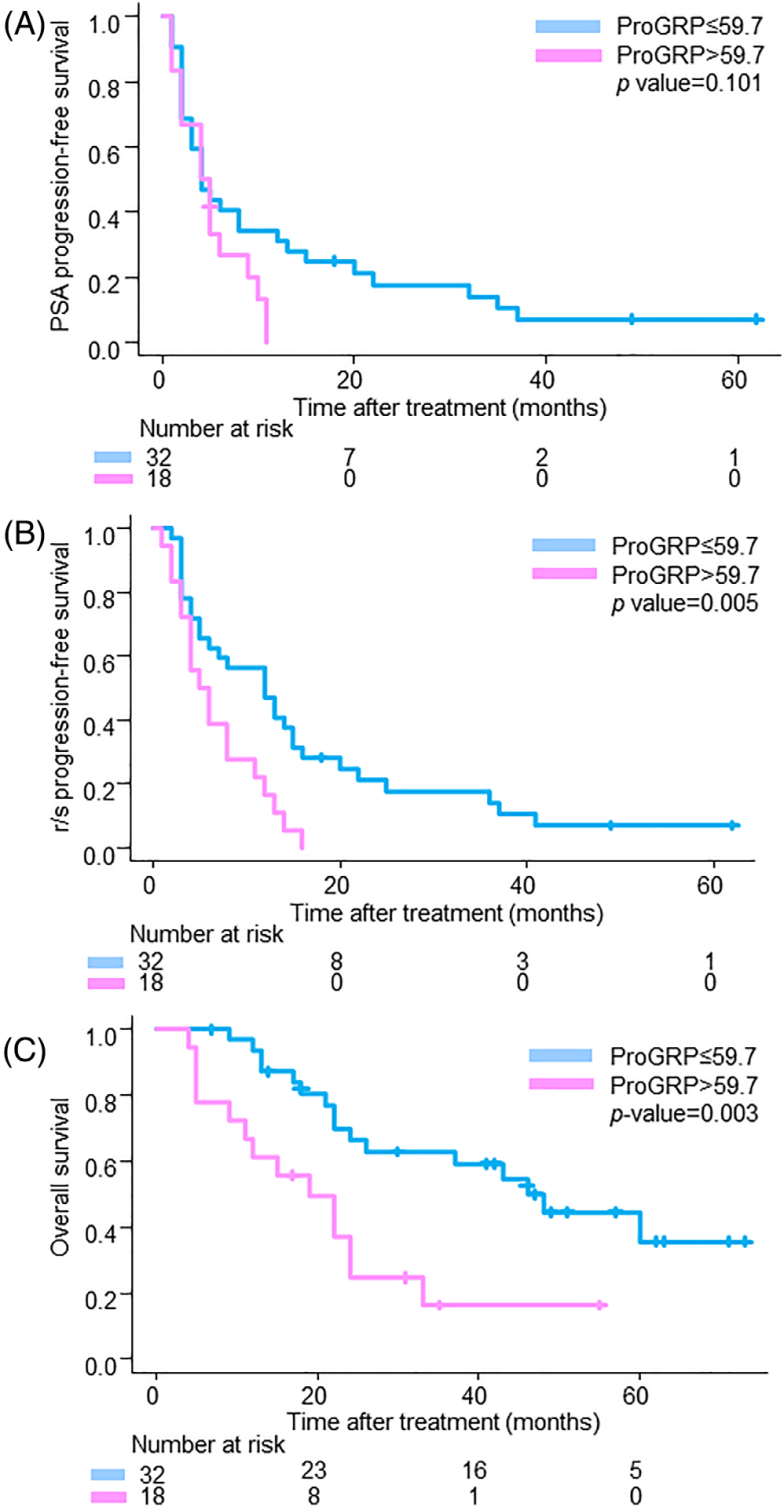
Kaplan–Meier curves of survival outcomes for metastatic CRPC patients who underwent treatment with abiraterone acetate/prednisone (A: PSA‐PFS, B: r/s PFS, C: OS).

After a median follow‐up of 24.0 months, 46 (92.0%) patients were interpreted as having PSA progression, 47 (94.0%) patients were diagnosed as having r/s progression, and 30 (60.0%) patients had died. ProGRP did not predict PSA‐PFS (Figure 3A), and only the neutrophil‐to‐lymphocyte ratio (NLR) was a statistically significant factor for PSA‐PFS (Table 5). In analyses of r/s PFS, ProGRP was a significant factor in the univariate analysis but lost its independent predictive value in the multivariate analysis (HR 1.89, 95% CI 0.93–3.83, *p* = 0.078). The NLR and duration of primary ADT sustained an independent predictive value (Figure 3B, Table 6). In analyses of OS, the univariate analysis showed that ProGRP, ECOG PS, hemoglobin, PSA, duration of primary ADT, and interval from the initial diagnosis to AA/P administration were statistically significant factors. The multivariate analyses showed that ProGRP, duration of primary ADT, and ECOG‐PS sustained an independent predictive value (ProGRP: HR 2.44, 95% CI 1.07–5.55, *p* = .034) (Figure 3C, Table 7).

**TABLE 5:** Factors associated with PSA progression‐free survival after treatment with AA/P

		Univariate			Multivariate^a^		
Factor	Category (*n*)	HR	95% CI	*p*‐value	HR	95% CI	*p*‐value
Age (years)	>77 (17) vs. ≤77 (33)	1.863	0.966–3.593	.063	—	—	—
ECOG PS 0/≥1	≥1 (14) vs. 0 (36)	1.481	0.782–2.803	.228	—	—	—
Hemoglobin (g/dl)	≤12.1 (20) vs. >12.1 (30)	1.339	0.734–2.440	.341	—	—	—
NLR ratio	>2.7 (24) vs. ≤2.7 (26)	2.708	1.397–5.248	.003	2.561	1.309–5.010	.006
Testosterone (ng/dl)	≤3.0 (32) vs. >3.0 (18)	1.137	0.594–2.176	.700	—	—	—
PSA (ng/ml)	>14.6 (21) vs. ≤14.6 (29)	1.442	0.785–2.650	.238	—	—	—
ALP (IU/l)	>284 (29) vs. ≤284 (21)	0.899	0.498–1.623	.724	—	—	—
NSE (ng/ml)	>12.4 (21) vs. ≤12.4 (29)	0.882	0.480–1.620	.686	—	—	—
ProGRP (pg/ml)	>59.7 (18) vs. ≤59.7 (32)	1.643	0.861–3.136	.132	—	—	—
Duration 1st ADT (month)	≤15.5 (23) vs. >15.5 (27)	1.513	0.832–2.750	.175	—	—	—
Dx to AA/P (month)	≤34.0 (26) vs. >34.0 (24)	1.046	0.585–1.873	.879	—	—	—
Prior docetaxel	yes (12) vs. no (38)	1.500	0.754–2.985	.248	—	—	—
Prior ENZ	yes (17) vs. no (33)	1.913	1.023–3.581	.042	1.609	0.829–3.122	.160
Metastatic burden	Bone+ (22) vs. Bone only (28)	0.967	0.536–1.745	.911	—	—	—

^a^ Analysis model consisted of appropriate factors.

Abbreviations: AA/P, abiraterone acetate/prednisone; ADT, androgen‐deprivation therapy; ALP, alkaline phosphatase; CI, confidence interval; Dx, diagnosis; ECOG PS, Eastern Cooperative Oncology Group performance status; ENZ, enzalutamide; HR, hazard ratio; NLR, neutrophil to lymphocyte ratio; NSE, neuron‐specific enolase; ProGRP, progastrin‐releasing peptide; PSA, prostate‐specific antigen; vs, versus.

**TABLE 6:** Factors associated with r/s progression‐free survival after treatment with AA/P

		Univariate			Multivariate^a^		
Factor	Category (*n*)	HR	95% CI	*p*‐value	HR	95% CI	*p*‐value
Age (years)	>77 (17) vs. ≤77 (33)	2.119	1.109–4.049	.023	—	—	—
ECOG PS 0/≥1	≥1 (14) vs. 0 (36)	1.529	0.814–2.872	.187	—	—	—
Hemoglobin (g/dl)	≤12.1 (20) vs. >12.1 (30)	1.601	0.884–2.900	.121	—	—	—
NLR ratio	>2.7 (24) vs. ≤2.7 (26)	3.193	1.608–6.340	<.001	2.977	1.481–5.983	.002
Testosterone (ng/dl)	≤3.0 (32) vs. >3.0 (18)	1.006	0.525–1.926	.986	—	—	—
PSA (ng/ml)	>14.6 (21) vs. ≤14.6 (29)	1.684	0.926–3.066	.088	0.953	0.472–1.924	.892
ALP (IU/l)	>284 (29) vs. ≤284 (21)	1.026	0.574–1.834	.932	—	—	—
NSE (ng/ml)	>12.4 (21) vs. ≤12.4 (29)	0.939	0.516–1.708	.867	—	—	—
ProGRP (pg/ml)	>59.7 (18) vs. ≤59.7 (32)	2.332	1.233–4.410	.009	1.888	0.931–3.827	.078
Duration 1st ADT (month)	≤15.5 (23) vs. >15.5 (27)	2.033	1.122–3.684	.019	2.029	1.066–3.861	.031
Dx to AA/P (month)	≤34.0 (26) vs. >34.0 (24)	1.280	0.719–2.279	.401	—	—	—
Prior docetaxel	yes (12) vs. no (38)	1.324	0.669–2.622	.420	—	—	—
Prior ENZ	yes (17) vs. no (33)	1.408	0.767–2.588	.270	—	—	—
Metastatic burden	Bone+ (22) vs. Bone only (28)	0.927	0.517–1.661	.799	—	—	—

^a^ Analysis model consisted of appropriate factors.

Abbreviations: AA/P, abiraterone acetate/prednisone; ADT, androgen‐deprivation therapy; ALP, alkaline phosphatase; CI, confidence interval; Dx, diagnosis; ECOG PS, Eastern Cooperative Oncology Group performance status; ENZ, enzalutamide; HR, hazard ratio; NLR, neutrophil to lymphocyte ratio; NSE, neuron‐specific enolase; ProGRP, progastrin‐releasing peptide; PSA, prostate‐specific antigen; r/s, radiographic and/or symptomatic; vs, versus.

**TABLE 7:** Factors associated with overall survival after treatment with AA/P

		Univariate			Multivariate^a^		
Factor	Category (*n*)	HR	95% CI	*p*‐value	HR	95% CI	*p*‐value
Age (years)	>77 (17) vs. ≤77 (33)	1.980	0.933–4.202	.075	—	—	—
ECOG PS 0/≥1	≥1 (14) vs. 0 (36)	2.530	1.188–5.386	.016	3.092	1.147–8.336	.026
Hemoglobin (g/dl)	≤12.1 (20) vs. >12.1 (30)	2.435	1.178–5.030	.016	—	—	—
NLR ratio	>2.7 (24) vs. ≤2.7 (26)	1.966	0.914–4.214	.082	1.026	0.431–2.442	.955
Testosterone (ng/dl)	≤3.0 (32) vs. >3.0 (18)	0.836	0.368–1.899	.668	—	—	—
PSA (ng/ml)	>14.6 (21) vs. ≤14.6 (29)	3.930	1.853–8.333	<.001	2.248	0.934–5.410	.071
ALP (IU/l)	>284 (29) vs. ≤284 (21)	0.850	0.414–1.745	.658	—	—	—
NSE (ng/ml)	>12.4 (21) vs. ≤12.4 (29)	1.525	0.723–3.216	.268	—	—	—
ProGRP (pg/ml)	>59.7 (18) vs. ≤59.7 (32)	2.984	1.404–6.344	.004	2.437	1.069–5.553	.034
Duration 1st ADT (month)	≤15.5 (23) vs. >15.5 (27)	2.653	1.269–5.544	.009	2.597	1.051–6.413	.039
Dx to AA/P (month)	≤34.0 (26) vs. >34.0 (24)	2.213	1.056–4.638	.035	—	—	—
Prior docetaxel	yes (12) vs. no (38)	1.031	0.441–2.410	.945	—	—	—
Prior ENZ	yes (17) vs. no (33)	0.843	0.383–1.852	.670	—	—	—
Metastatic burden	Bone+ (22) vs. Bone only (28)	1.451	0.706–2.982	.311	—	—	—

^a^ Analysis model consisted of appropriate factors.

Abbreviations: AA/P, abiraterone acetate/prednisone; ADT, androgen‐deprivation therapy; ALP, alkaline phosphatase; CI, confidence interval; Dx, diagnosis; ECOG PS, Eastern Cooperative Oncology Group performance status; ENZ, enzalutamide; HR, hazard ratio; NLR, neutrophil to lymphocyte ratio; NSE, neuron‐specific enolase; ProGRP, progastrin‐releasing peptide; PSA, prostate‐specific antigen; r/s, radiographic and/or symptomatic; vs, versus.

### 3.3 Impact on results of ProGRP and PSA dynamics

The rate of changes in ProGRP levels at 6 months from baseline or at event occurrence in the ENZ and AA/P series ranged from −35.8% to 332% and −35.8% to 135%, respectively. Analyses were also performed between groups with significantly increased or those who maintained high ProGRP levels (*n* = 24 in the ENZ series and *n* = 20 in the AA/P series) and decreased or those who maintained low ProGRP levels (*n* = 26 in the ENZ series and *n* = 30 in the AA/P series), rather than between groups simply divided by the rate of increase or decrease of ProGRP levels. Five (10%) and 6 (12%) patients were regrouped by the ProGRP dynamics classification for the ENZ and AA/P series, respectively.

In the ENZ series, ProGRP dynamics correlated with the maximum PSA change from baseline (increased or maintained a high level of ProGRP: −34.5% vs. decreased or maintained a low level of ProGRP: −78.4%, *p* = .048), and showed independent predictive values for all survival estimates. In the AA/P series, ProGRP dynamics did not correlate with the maximum PSA change from baseline (increased or maintained a high level of ProGRP: −21.7% vs. decreased or maintained a low level of ProGRP: −36.9%, *p* = .415) or PSA‐PFS, whereas they showed an independent predictive value for r/s PFS and OS.

Even when the PSA dynamics observed duringthe treatment with ARAT agents were added to their multivariate model, ProGRP dynamics showed independent predictive values for r/s PFS and OS in both ARAT agent series, while PSA dynamics could not hold independent predictive values for OS in the AA/P series.

## 4 DISCUSSION

The significance of the NE pathway and the measurement of blood NE markers in patients with metastatic PCa have become recognized over the last two decades.^11^, ^17^ ProGRP, a region common to three different subtypes of GRP precursors, is a biomarker detectable in the blood due to its longer half‐life than GRP itself and is biologically active as a growth factor. Moreover, ProGRP values show excellent sensitivity and correlation with therapeutic response in small‐cell lung carcinoma compared to the classic tumor markers.^14^ Our research group first reported that serum ProGRP is a useful biomarker for monitoring clinical course in small‐cell NE PCa and also for assessing the NE environment and prognosis in conventional metastatic and hormone‐resistant PCa.^18^, ^19^, ^20^, ^21^ Since that report, serum/plasma ProGRP has been evaluated in our routine clinical practice in metastatic PCa patients at initial diagnosis and clinical events, leading to a prospective estimation of the NE environment and the determination of treatment strategies.

In the present study, plasma ProGRP provided a consistent predictive value for OS in metastatic CRPC patients who underwent treatments with ARAT agents. On the other hand, ProGRP unexpectedly showed a different predictive profile between the two ARAT agents in the therapeutic response assessed by PSA change and its long‐lasting effects. ProGRP predicted PSA dynamics when treated with ENZ, but not with AA/P. The information obtained from ProGRP dynamics was similar to that of single‐point measurement, but with the additional consideration that it is an independent predictor of r/s PFS in the AA/P series. Furthermore, ProGRP dynamics showed an advantage in the predictive value of OS during AA/P treatment compared to PSA dynamics.

These findings suggest that ProGRP is a promising biomarker in CRPC patients and that GRP may be a therapeutic target. Previous reports on the link between GRP and AR signaling pathways, or the link between GRP and CRPC progression, are limited in both clinical and basic research fields.^12^, ^13^, ^22^, ^23^, ^24^, ^25^ Therefore, this study adds to current research with its observations on these relationships from a clinical aspect through the treatment outcomes with ARAT agents.

Chromogranin‐A (CgA) and NSE are both NE markers that have been investigated in serum and histopathology.^26^, ^27^ A direct comparison between them revealed that CgA was an excellent indicator in the correlation between serum levels and tissue immunostaining,^28^, ^29^ and the predictive value of OS at those serum levels was equivalent to or slightly better for CgA than for NSE.^30^, ^31^, ^32^, ^33^ The prognostic value of serum/plasma ProGRP for OS should also be definitive, considering our previous report and that of an independent research group.^21^, ^34^ However, serum NSE did not consistently provide any predictive information in our previous analyses,^21^ or in this study. Unfortunately, our study did not include serum CgA and a comparison with ProGRP was not possible. The same is true for other neuropeptides, but GRP resources are not exclusively restricted to prostate NE tissue.^35^ Moreover, the concordance of plasma and tissue expression has not been investigated except for small‐cell NE carcinoma. To date, there are fewer than 20 reports on PCa and serum/plasma ProGRP, including ours. Thus, ProGRP is positioned as a NE marker with immature information compared to CgA in PCa.

Interestingly, ProGRP showed a link with PSA response and PSA‐PFS in patients treated with ENZ. Similarly, in our previous report, in which the patient was treated with ADT and classic anti‐androgens, we already suggested such findings.^20^ In contrast, studies on CgA or NSE have shown little association with PSA response, except for PSA‐PFS and r/s PFS when these two NE markers are complementary.^32^, ^33^, ^36^ Although the results might depend on a combination of the NE markers and treatment modality, it is not possible to address the mechanism underlying the difference in predictive profiles of ProGRP between ENZ and AA/P without supporting evidence. Furthermore, CRPC often has a weak relationship between the surrogate endpoint and the OS as the true endpoint,^37^ therefore, this finding also needs to be reproduced in a validation cohort to characterize ProGRP.

Current knowledge of molecular mechanisms is limited, and discussions beyond speculation are topics left for future research. Below, we list some issues to be identified, each of which has different feasibility in basic and clinical fields. First, the nature of NE markers, including ProGRP, should be comprehensively investigated in a sufficient population to determine the degree of involvement in CRPC progression. Although there are reports examining NE markers during docetaxel chemotherapy and during the treatment with ARAT agents,^25^, ^33^ the determination of the predictive value of NE markers across different treatments and disease stages, such as CRPC or earlier, is still inadequate. In addition, the mechanisms resistant to ARAT agents should be further investigated. Currently, there are candidate mechanisms common to ARAT agents that lead to cross‐resistance as well as those specific to each ARAT agent due to different mechanisms of action.^38^, ^39^, ^40^ However, no studies have investigated the relevance of GRP to the above mechanisms, especially to specific ones. As GRP has two aspects, one as a non‐AR‐associated factor produced from NE tissue and the other as an AR‐associated factor revealed by basic research, its involvement in the treatment resistance is expected to be complex.

The results of this study raise a unique approach to guide treatment with ARAT agents by referencing ProGRP values. As a premise, this study was not designed to compare survival outcomes by treatment sequences and is, therefore, limited to the selection of potentially beneficial ARAT agents. At least, in cases with high ProGRP levels, it may be better to avoid starting treatment with ENZ. In addition, treatment modalities with other targets should be considered. It has been reported that ARAT preceded by docetaxel improved prognosis when NE markers were elevated by the complementary judgment of CgA and NSE.^41^ Moreover, a randomized crossover trial found that treatment with AA/P followed by ENZ was the optimal sequencing for the clinical benefit.^42^ A similar result was also reported in Japan,^43^ and an acquired alteration of the AR signaling axis was postulated as the mechanism. However, if our findings are validated, the involvement of the NE pathway via GRP cannot be excluded either.

Although data were collected prospectively, several limitations restrict the quality of the results. The study is a retrospective analysis conducted at a single institution with nonrandomized administration of ARAT agents and short follow‐up periods. Importantly, this study did not include a true control group as most patients resistant to one ARAT agent will also be resistant to another. Therefore, as well as a need for a larger validation cohort, a CRPC series treated with concomitant drugs without cross‐resistance to ARAT agents or an ADT‐only series is necessary as a control to confirm the effect of ProGRP level on treatment outcomes. Because there is evidence of a favorable prognosis for treatment with ARAT agents, we could not include an independent control group at this time in real clinical practice.

Despite such limitations and deficiencies, this report provides a valuable suggestion on how the NE pathway is involved in CRPC progression via GRP and possible differences in the effects of ENZ and AA/P treatment.

## 5 CONCLUSIONS

Plasma ProGRP provides a consistent predictive value for OS in metastatic CRPC patients who underwent ARAT agents. Meanwhile, ProGRP showed different predictive profiles for PSA‐ and r/s PFS between ENZ and AA/P treatment. These findings clinically suggest a mechanism for CRPC progression involving the NE pathway via GRP, however, the underlying mechanism of different predictive profiles by ARAT agents requires future research. Therefore, therapeutic strategies for metastatic CRPC should be directed toward deterring the NE pathway as well as targeting the AR‐axis.

## AUTHOR CONTRIBUTIONS

**Masahiro Yashi:** Conceptualization (lead); formal analysis (lead); methodology (lead); project administration (lead); writing – original draft (lead); writing – review and editing (lead). **Daisaku Nishihara:** Data curation (lead); methodology (equal); writing – review and editing (supporting). **Megumi Yokoyama:** Data curation (equal); formal analysis (supporting). **Hirotaka Fuchizawa:** Data curation (supporting); formal analysis (supporting). **Akihito Okazaki:** Data curation (supporting); formal analysis (supporting). **Kohei Takei:** Formal analysis (supporting); methodology (supporting). **Issei Suzuki:** Formal analysis (supporting); methodology (supporting). **Kazumasa Sakamoto:** Data curation (supporting); methodology (supporting). **Toshiki Kijima:** Conceptualization (supporting); supervision (supporting). **Minoru Kobayashi:** Supervision (supporting). **Takao Kamai:** Project administration (supporting); supervision (lead).

## CONFLICT OF INTEREST

The authors declare that they have no competing interests.

## Data Availability

The authors declare that informed consent was obtained for the publication of patient data. The datasets used and/or analyzed during the current study are available from the corresponding author on reasonable request.
